# Community-embedded follow-up management intervention for geriatric primary care: a mixed-methods study of an integrated health services model

**DOI:** 10.1186/s12913-024-10804-8

**Published:** 2024-03-06

**Authors:** Wenjing Shi, Lingling Wu, Xiaodong Li, Feng Qi, Wanyu Ji

**Affiliations:** 1 Xinglin College, Nantong University, 226019 Nantong, China; 2 Department of Orthopedics, The Yancheng Clinical College of Xuzhou Medical University (The First People’s Hospital of Yancheng), 224001 Yancheng, China; 3 School of Public Health, Nantong University, 226019 Nantong, China; 4 Department of Pharmacy, The Yancheng Clinical College of Xuzhou Medical University (The First People’s Hospital of Yancheng), 224001 Yancheng, China

**Keywords:** Older adults, Living alone, Community services, Osteoporosis, Professional support

## Abstract

**Background:**

To propose a community-embedded follow-up management model to provide health services for elderly patients with osteoporosis who live alone.

**Methods:**

Researchers randomly selected 396 people with osteoporosis living alone from five communities in Nantong, China, for the study. These participants were randomly assigned to control and intervention groups. Twenty-four community physicians in five communities provided professional support based on a community-embedded follow-up management model. Participants completed quantitative questionnaires at baseline and after the 6-month follow-up intervention, and some participants underwent semi-structured face-to-face interviews. The primary outcome is the effectiveness of the community-embedded follow-up management model in improving the quality of life of elderly patients with osteoporosis living alone. Based on an objective quantitative assessment, the qualitative study explains and adds essential components of this community-based follow-up management model.

**Results:**

The quantitative study showed that scores in physical functioning, ability to perform daily activities, self-efficacy, and mental status were significantly improved in the intervention group compared to the control group (*p* < 0.05). The most significant improvements were found in “mental status” (*p* = 0.012) and “self-care skills” (*p* = 0.003). The qualitative study reported the essential elements of a community healthcare model for older people living alone with osteoporosis, including professional support, personalized services, social support, and empowerment.

**Conclusions:**

Community-embedded follow-up management meets the need for elderly patients with osteoporosis living alone. It helps to improve health perception, promote physical and mental health, and optimize the quality of life in this population. Personalized services and professional support are two major contributing factors to effective embedded follow-up management in the community.

**Supplementary Information:**

The online version contains supplementary material available at 10.1186/s12913-024-10804-8.

## Background

Demographics are changing around the world, and the global population is aging. In China, the proportion of people aged ≥ 65 years is increasing, and the population aged 65 years and older is expected to increase to approximately 400 million by 2050 [1]. Since the prevalence of osteoporosis increases with age, the number of elderly with osteoporosis is expected to increase [2, 3]. Osteoporosis is a systemic bone disease characterized by low bone mass and broken bone microarchitecture, leading to increased bone fragility and susceptibility to fracture [4]. The fragility fracture and post-fracture complications caused by this disease seriously affect the quality of life of the elderly population and even lead to death [5]. Studies have shown that living alone or transitioning from living with others significantly increases the risk of osteoporosis compared to non-living alone older adults [6, 7]. This may be due to a lack of socialization and social support, malnutrition, and poor psychological well-being [8–10].

The decline or loss of health status and ability to perform activities of daily living has increased their need for daily care, psychological comfort, and health management services [11]. However, most osteoporosis health care focuses on patients with recent or previous fractures (secondary prevention), with insufficient attention to individuals with no previous fractures. Older people living alone without a history of fractures are more likely to be neglected, and they tend to engage in routine self-care without professional support at the healthcare level [12, 13]. Most studies on integrated osteoporosis care models were implemented in the clinical phase and performed simultaneous assessment efforts [14]. However, there is an unmet need for health education, financial and staff resources, and increased collaboration and linkage between communities and physicians [15]. In particular, older adults living alone who lack social support have difficulty implementing timely coordination and updating of the health care delivery system they receive during comprehensive osteoporosis care [16].

As a frontline public health service team, community health workers (CHWs) have become essential supporters of health management services for older adults living alone. They integrate specific populations with health promotion and clinical services by providing medical and social care support services and being an essential player in connecting the healthcare system to the community [17, 18]. Many studies have proposed community health systems and community health worker programs have been proposed in many studies [19–21]. Agarwal et al. focused on effective policy, institutional, and implementation dimensions of CHWs and community health systems, reporting on the critical role of factors such as community embeddedness levels of CHWs and standardization of quality assessment of services in achieving the effectiveness of community health services [22]. He et al. examined the perceptions of health services for older adults in Singapore, Hong Kong, Malaysia, and Indonesia. The report found a significant trend toward integrating community-based long-term geriatric care with curative and preventive care [23].

In recent years, comprehensive geriatric community health management based on primary health care services has been used in geriatrics to develop treatment plans and predictive monitoring [24–26]. Specifically, the command includes mental health, exercise norms, and dietary management [27–29]. However, given the marked individual differences among older patients, how to deliver appropriate interventions in the community and the adaptability and effectiveness of interventions need to be further explored. Therefore, this study aimed to propose a community-embedded follow-up management model to provide health services for elderly patients with osteoporosis living alone, to evaluate the effectiveness of this community follow-up management model from the perspective of elderly patients with osteoporosis using mixed methods, to explore the perception of how the community can adequately intervene, and to seek a better community health service model to provide a comprehensive care environment for this population.

## Methods

### Study design and sampling

**Fig. 1:**
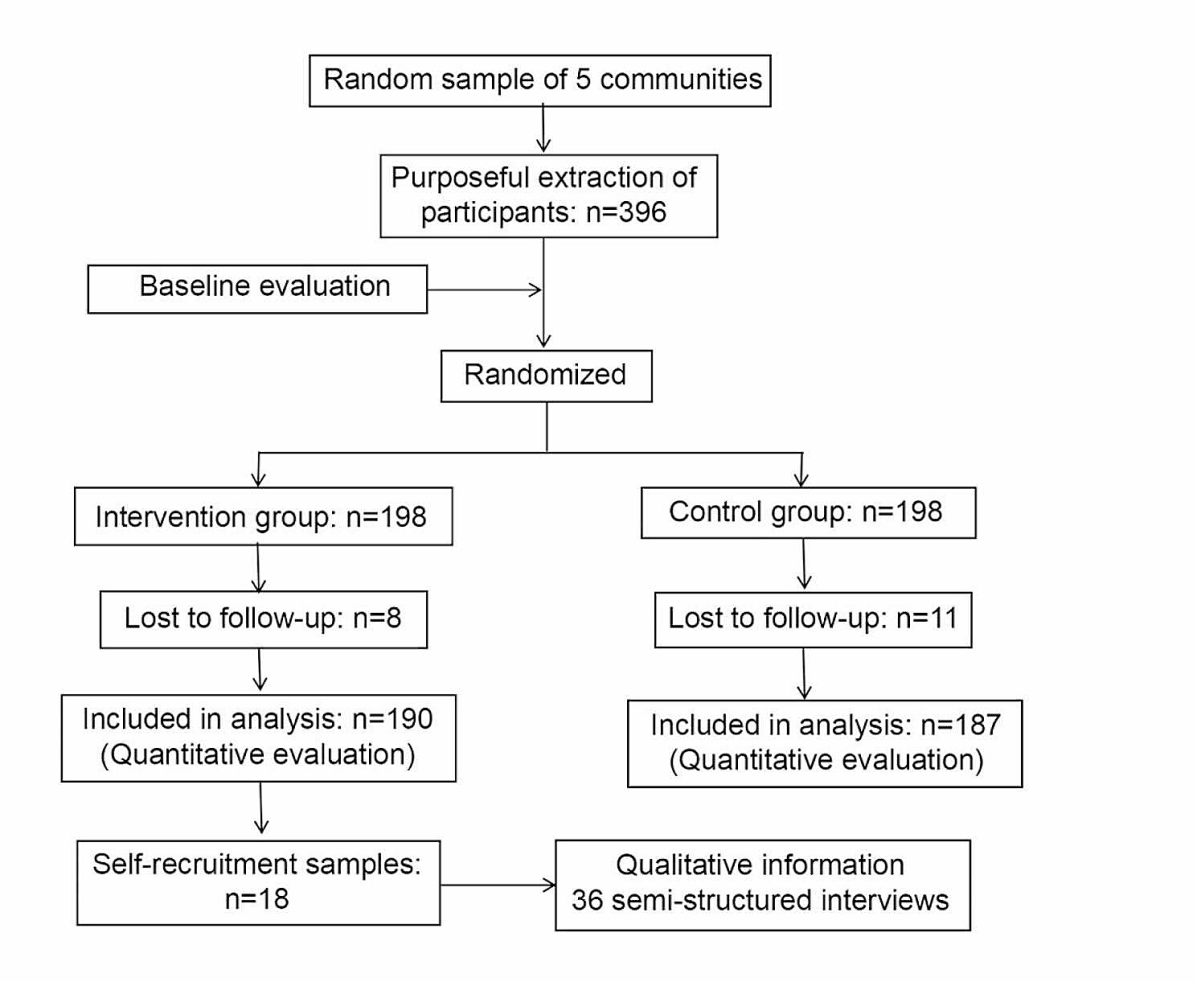
Flow chart of participants in the community-embedded follow-up intervention trial

This study used a mixed-methods approach combining quantitative and qualitative analyses to assess the effectiveness of a community-based embedded follow-up management model applied to the care of elderly patients with osteoporosis living alone (Fig. 1). The quantitative study component was a two-group randomized controlled trial. Participants were assigned to a control group (daily self-care) and an intervention group (community-embedded follow-up management). Based on an objective quantitative assessment, the qualitative study explains and adds essential components of this community-based follow-up management model. The Ethics Committee approved the study of the First People’s Hospital of Yancheng City [2022]-(K-030). The study was conducted between September 2021 and March 2022. The study was conducted using a random sampling method in Nantong, Jiangsu Province, China. Five communities were randomly selected from the area. One service site was set up in each community.

### Sample size

The study calculated the sample size using a one-sided test via PASS 2021 software. Based on the results of a pre-trial with a sample size of 30, the effectiveness of a community-embedded follow-up intervention for elderly patients with osteoporosis living alone improved by 20% compared to self-care outcomes. Considering a one-sided 2.5% significance level and a power of 95%, at least 150 study subjects were included in each intervention and control group [30]. The study ultimately enrolled 396 older adults with osteoporosis living alone in this study. The sample size for the intervention and control groups was 198 each.

### Inclusion and exclusion criteria

As implementers of this community-embedded follow-up intervention, community physicians from 5 communities were invited to participate in this study. After obtaining consent from 24 community physicians, they received standardized professional training. Community physicians would meet face-to-face with eligible participants in that community to inform general information about the study. Participant inclusion criteria: (1) living alone; (2) age ≥ 60 years; (3) meeting World Health Organization (WHO) diagnostic criteria for osteoporosis (based on dual-energy X-ray absorptiometry (DXA)); (4) no history of psychiatric disorders or cognitive impairment; (5) having normal communication and comprehension; (6) voluntary participation; (7) informed consent of the person or guardian.

Exclusion criteria: (1) comorbid mental illness or cognitive dysfunction; (2) severe liver and kidney dysfunction; (3) subjects unwilling to participate at any stage; (4) leaving the community; (5) serious illness resulting in long-term bed rest.

After obtaining written consent, participants completed quantitative questionnaires at baseline and after the 6-month follow-up intervention, and some participants underwent semi-structured face-to-face interviews.

### Intervention model

#### Preservation of autonomy

A focus on humanistic care is commonly emphasized in the aging care process. However, this element often results in varying degrees of constraint when it is overly reinforced. Such over-constraints are mainly reflected in (1) the denial of autonomy to older adults, (2) the severance of social ties, and (3) dignity [31]. A large and growing body of research reports the value of older adult empowerment in achieving self-care and helping older adults manage their health and care. Promoting self-care by empowering older adults can be effective in reducing the course of illness while maintaining the dignity of older adults [32]. Therefore, in this community intervention model, the design focused on the retention and support of autonomy for elderly patients with osteoporosis. This support is mainly reflected in incomplete interventions at patients’ cognitive and behavioral levels: (1) support for active learning of health knowledge; (2) guidance of healthy behavioral habits; (3) self-maintenance of social relationships.

#### Personalized services

**Fig. 2:**
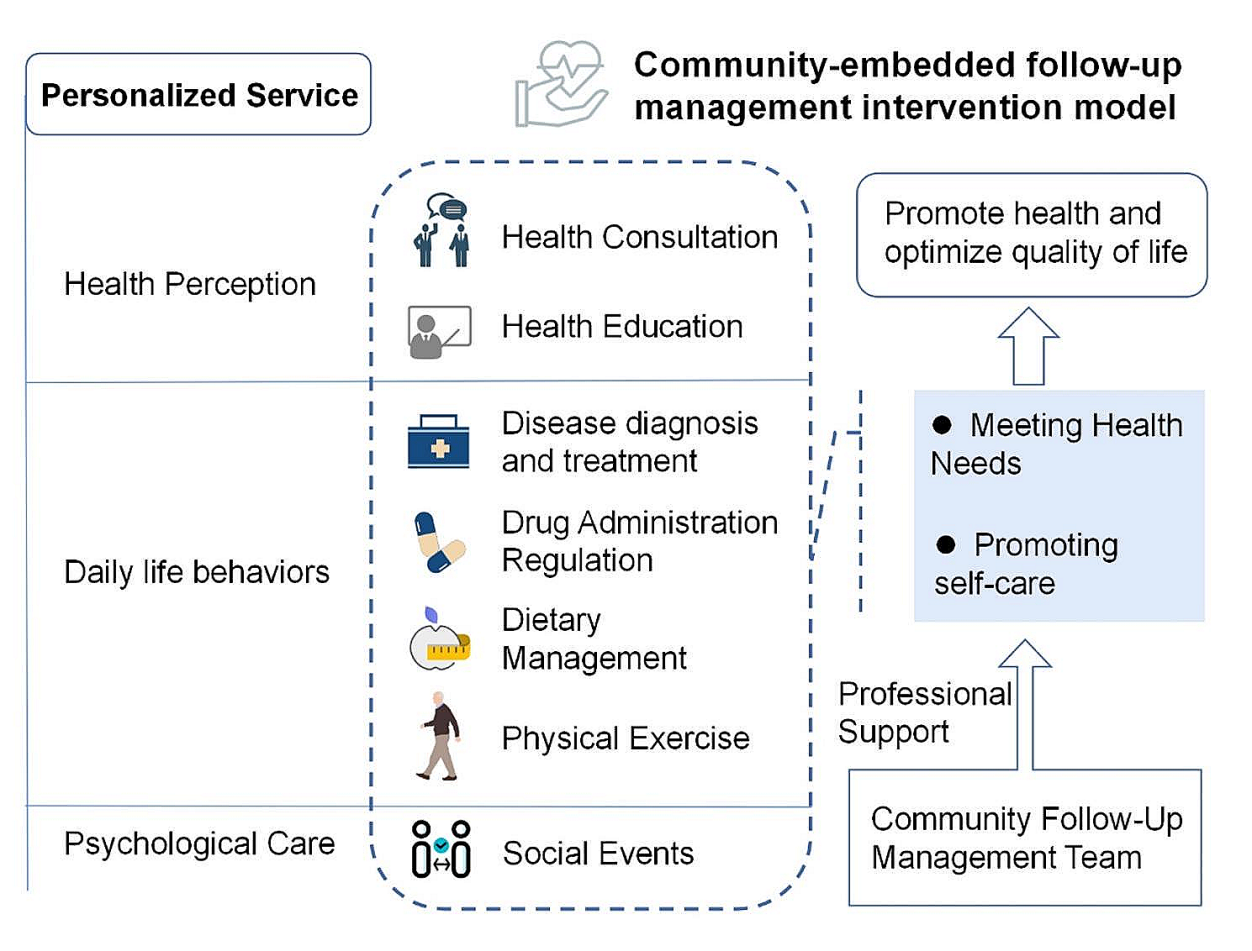
Community-embedded follow-up management intervention model

The primary goal of personalized services is to meet the health needs of different patients due to the significant variability between individuals. Personalized services are based on the Comprehensive Geriatric Assessment (CGA) exercise [33, 34]. The comprehensive geriatric assessment results are incorporated into the community intervention management questions and goals, and targeted aged osteoporosis follow-up management measures are implemented in terms of comprehensive medical care, nutritional diet, ability to perform daily living, cognitive ability, and psychology [35]. Although past research on senior care has examined characteristics of care patterns that affect different populations or individuals, a variety of possible health risk factors not only alter the community care setting of health care delivery in a broad range of ways but also increase uncertainty and personal risk. Given the limited social activities of elderly osteoporotic patients living alone, it is primarily a community-wide activity. Patients will need health care services in the community. Searching for personalized senior health care is a fundamental building block of this community follow-up management [36]. The advantages of this customized service are (1) prediction of health risks, (2) monitoring of disease progression dynamics, and (3) health care plans tailored to individual needs. Therefore, this follow-up intervention involves multiple levels of health education, condition monitoring, daily behavior guidance, and psychological care, and the specific intervention model is shown in Fig. 2; Table 1.

**Table 1:** Specific content and measures of community-embedded follow-up intervention

Projects	Specific measures
Dietary Modifications	A balanced diet rich in calcium, low in salt, and moderate in protein, avoiding irregularity.
	Quit smoking
	Restrictions on alcohol consumption
	Avoid excessive consumption of coffee and carbonated beverages.
Exercise instruction	Appropriate outdoor activities (regular muscle strength exercises) and sun exposure, such as walking and other low-intensity exercise (tai chi, dance, table tennis).
Health Education	Personal health coaching, 30 min, 2 times/week;
	Intensive health education, 120 min, 1 time/month
Medical treatment and medication	Daily diagnosis and monitoring of chronic diseases and other basic diseases;
	Bone Density Health Status Scale - International Osteoporosis Foundation (IOF) Minute Test of Osteoporosis Risk, every three months;
	Medication for the underlying disease and/or combination of osteoporosis symptomatic drugs: calcium, vitamin D, etc.
Psychological support care	Encourage social interaction activities, such as peer education, 1 time/week;
	Telephone and home follow-up visits, 2 times/week

Considering the participants’ acceptance and familiarity with their respective community settings, local community physicians, nurses, and community senior caregivers formed the follow-up intervention teams in their communities. They all received standardized training in osteoporosis and health-related behaviors and had organizational and coordination skills. At the beginning of the intervention, community physicians and nurses conducted an evidence-based assessment. Based on the comprehensive assessment results, they developed an individualized intervention plan for older patients with osteoporosis who lived alone. The intervention plan was dynamically adjusted accordingly to the assessment results during the intervention. Community geriatric care providers were responsible for monitoring, guiding, and assisting in completing the intervention.

### Quantitative research component

#### Study design

Considering the specificity of the study population and intervention mode, the study used a questionnaire designed by a professional team to collect quantitative data. The quality of the questionnaire was tested for reliability and validity through pre-testing [37]. A Cronbach alpha internal consistency method with a sample of 100 was used. The reported coefficients of ability to perform daily activities were α = 0.86, self-efficacy α = 0.81, mental status α = 0.78, and satisfaction assessment α = 0.87.

The first questionnaire assessment was conducted in the initial period of the study, in September 2021. The questionnaire was completed again six months after the community-embedded follow-up management intervention. The aim is to provide an objective evaluation of the effectiveness of the community-embedded follow-up management model applied to the health care of elderly osteoporosis patients living alone.

The questionnaire covered five dimensions: physical function, ability to perform daily life, psychological status, self-efficacy, and satisfaction. Among them, pain symptoms and frailty were the most intuitive indicators of physiological function changes. Pain symptoms and frailty were assessed visually based on the Visual analogue scale (VAS) pain rating scale. Participants rated pain symptoms and frailty on a scale of 1 (weak) to 10 (firm). Higher scores suggest a poorer physiological functional status. The other sections consisted of Likert scales and judgmental entries. Higher scores indicate higher quality of life and benefit assessment. Specific indicators for assessing the effectiveness of the application of the Community Embedded Follow-up Management model are shown in Table 2.

**Table 2:** Indicators for assessing the effectiveness of the application of community-embedded follow-up management model

Projects		Number of items (Format)	Scoring Criteria (Range)
Physiological functions			2–20
	Pain Symptoms		1–10: Weak - Strong (1–10)
	Degree of weakness		1–10: Weak - Strong (1–10)
Ability to perform daily life			20–100
	Social Behavior	8 items / 5-point Likert scale (strongly disagree to strongly agree)	Strongly disagree = 1, Disagree = 2, Don’t know = 3, Agree = 4, Strongly agree = 5 (8–40)
	Diet rules	6 items / 5-point Likert scale (strongly disagree to strongly agree)	Strongly disagree = 1, Disagree = 2, Don’t know = 3, Agree = 4, Strongly agree = 5 (6–30)
	Exercise Capacity	6 items / 5-point Likert scale (strongly disagree to strongly agree)	Strongly disagree = 1, Disagree = 2, Don’t know = 3, Agree = 4, Strongly agree = 5 (6–30)
Self-efficacy			15–105
	Health Awareness	15 items (True - False - Don’t know)	“Correct” response = 2, “Don’t know” response = 1, “Incorrect” response = 0 (0–30)
	Self-care responsibility	7 items / 5-point Likert scale (strongly disagree to strongly agree)	Strongly disagree = 1, Disagree = 2, Don’t know = 3, Agree = 4, Strongly agree = 5 (7–35)
	Self-care skills	8 items / 5-point Likert scale (strongly disagree to strongly agree)	Strongly disagree = 1, Disagree = 2, Don’t know = 3, Agree = 4, Strongly agree = 5 (8–40)
Mental state		8 items / 5-point Likert scale (strongly disagree to strongly agree)	Strongly disagree = 1, Disagree = 2, Don’t know = 3, Agree = 4, Strongly agree = 5 (8–40)
Satisfaction Assessment		12 items / 5-point Likert scale (strongly disagree to strongly agree)	Strongly disagree = 1, Disagree = 2, Don’t know = 3, Agree = 4, Strongly agree = 5 (12–60)

#### Randomization and blinding

The study used the random number generator built into SPSS 26.0 to perform covert and digital randomization groupings. Participants were assigned with equal probability to either number 1 or 2. Number 1 was the control group, where participants did not receive any intervention during the study period. Number 2 was the intervention group, where participants received health services in a community-embedded follow-up management model.

#### Data analysis

The study used SPSS 26.0 statistical software to process the quantitative data. Count data were described by counts ( n ) and proportions (%). The mean and standard deviation indicate the trend of the sample set. Kolmogorov-Smirnov tested the normality of the data. Since the data did not conform to a normal distribution, a nonparametric test was used. Differences between groups were tested using the Mann-Whitney U and chi-square tests for categorical variables. The Wilcoxon Signed Ranks Test examined differences in physical functioning, daily living abilities, psychological status, self-efficacy, and satisfaction of participants before and after the intervention. The level of statistical significance was set at less than 0.05.

### Qualitative research section

#### Measures

The interview study aimed to explore the experiences of health services for older people with osteoporosis living alone and to assess the group’s perceptions of effective implementation of community-embedded follow-up management. The 18 respondents who participated in the interviews were from 190 participants in the quantitative research intervention group. After receiving six months of community-embedded follow-up intervention services, they were randomly selected to participate in this qualitative interview study. Among them, refusal or withdrawal from the interview at any stage was considered an exclusion criterion for the qualitative research. An interview research team of six experienced qualitative researchers was formed. The study collected data through in-depth face-to-face interviews and follow-up observations recorded by the researcher with each participant until no other potentially qualitative data were available. To ensure the completeness and accuracy of the qualitative research data, each participant was interviewed twice in duplicate, meaning that the same interview guide was followed for both interviews. Therefore, 36 face-to-face semi-structured interviews were conducted for the qualitative study. Interviews were born in the participants’ respective homes. The participants themselves scheduled interviews, and each interview lasted between 30 and 40 min. The researcher conducted interviews after obtaining consent from the participants. Qualitative interview guides were designed by professional staff. According to the construction focus of the community-embedded follow-up management model and the assessment points of the quantitative study, the qualitative interview guide for the respondents included (1) physical condition, (2) daily activity status, and (3) perceptions of community-embedded follow-up management. A researcher recorded the interviews. The study ensured the quality of the discussions and transcriptions by listening to the audio-recorded interviews and reading the follow-up transcripts for simultaneous analysis.

#### Data analysis

The study analysis used Strauss’s three-process coding method. The research involved open coding, spindle coding, and selective coding. Two groups of experienced qualitative researchers were responsible for analyzing and reviewing the data, respectively. Initial data analysis was performed using N-Vivo 12 for open coding, and data were categorized and dimensionalized using subheadings according to interview guidelines. The concepts of relevant categories were organized inductively into a sequence that constituted the axial coding. The researchers created condition matrices based on the axial coding paradigm. The matrix helped achieve a visual analysis of the conditions and thus selectively coded to generate themes. Finally, another group of researchers further reviews the data, commenting and changing the themes. The reliability and accuracy of the assessment results are ensured.

## Results

### Demographic information

The total sample size at baseline for the quantitative study was 396 cases (Table 3). The sample size for the intervention and control groups was 198 each. There were no significant differences between groups in the baseline characteristics of the intervention and control groups. During the study period, 19 (4.80%) dropped out and shed at follow-up, including 11 in the control group and 8 in the intervention group. The investigator guided participants to complete the questionnaire thoroughly and accurately to ensure no missing values in the quantitative data.

Eighteen participants were interviewed for the qualitative study. Eleven of them were female, and seven were male. The mean age was 66.89 years (SD = 4.06).

**Table 3:** Basic information of the participants (*N* = 396)

Variable		Control	Intervention	*P*-value*
		*n* = 198	*n* = 198	
Gender,n(%)				0.510
	Male	108(54.5)	101(51.0)	
	female	90(45.5)	97(49.0)	
Gender, mean (SD)		1.45(0.50)	1.49(0.50)	
Age,n(%)				0.399
	60–64	51(25.8)	55(27.8)	
	65–69	89(44.9)	71(35.9)	
	70–74	55(27.8)	64(32.3)	
	≥ 75	3(1.5)	8 (4)	
Clinical Features,n(%)				
	Hypertension	36(18.2)	44(22.2)	0.317
	Hypercholesterolemia	13(6.6)	23(11.6)	0.081
	Diabetes	17(8.6)	25(12.6)	0.192
	Heart disease	10(5.1)	18(9.1)	0.117
	Stroke	5(2.5)	11(5.6)	0.126
	Chronic lung disease	27(13.6)	32(16.2)	0.481
	Fall in the past year	42(21.2)	49(24.7)	0.404
	Hip fracture in the past year	5(2.5)	4(2.0)	0.736
	Non-femoral fracture in the past year	29(14.6)	34(17.2)	0.493
	≥ 5 medications at screening	8(4.0)	16(8.1)	0.092

SD = standard deviation

*Mann-Whitney Test

### Quantitative results

Table 4 shows that the intervention and control groups achieved favorable results regarding physical status scores, daily living skills, self-efficacy, and psychological status. Compared to the control group, the intervention group showed significant improvement in the scores of all dimensions. The most significant improvements were observed in the areas of “Mental state” (*p* = 0.012) and “self-care skills” (*p* = 0.003). Participants’ satisfaction with the healthcare services provided under this model increased significantly.

**Table 4:** Intervention effects of community-embedded follow-up management model

Outcome	Intervention (*n* = 190)		Control (*n* = 187)		*P* value^*^
	Baseline	Follow-up	Baseline	Follow-up	
Pain level, mean (SD)	6.29(0.91)	4.72(0.93)	6.39(0.95)	6.26(1.17)	<0.001
Degree of weakness, mean (SD)	5.87(1.11)	4.25(1.06)	6.01(1.045)	5.64(1.30)	0.010
Social behavior score, mean (SD)	20.78(1.74)	32.61(4.31)	20.86(1.68)	23.06(5.37)	0.003
Dietary score, mean (SD)	14.31(1.57)	24.16(3.02)	14.41(1.72)	16.30(4.32)	<0.001
Exercise capacity Score, mean (SD)	14.18(1.27)	25.90(3.32)	14.28(1.31)	16.48(4.89)	0.001
Mental state, mean (SD)	18.60(1.65)	32.91(3.99)	18.70(1.78)	21.33(6.20)	0.012
Health awareness level, mean (SD)	19.77(4.16)	26.17(1.93)	19.96(4.24)	21.02(4.69)	0.011
Self-care responsibility, mean (SD)	18.93(2.26)	25.50(2.73)	18.74(2.13)	20.30(3.50)	<0.001
Self-care skills, mean (SD)	20.53(1.54)	33.27(3.23)	20.57(1.59)	22.95(5.37)	0.003
Satisfaction, mean (SD)	27.23(2.37)	51.45(5.92)	27.26(2.39)	31.71(10.31)	0.026

SD = standard deviation

*Wilcoxon signed-rank test

### Qualitative findings

The majority of participants reported that community-embedded follow-up management services met their health care needs. The satisfaction from this management intervention was mainly in health perceptions, daily behavioral outcomes, and psychological care.

### Health Awareness

Participants acknowledged the benefits of community-embedded follow-up management in terms of health awareness, helping them improve their health awareness [18]. They valued the ability to access more detailed information in an easy-to-understand format during the management process. This would meet their need for information while living alone.

“…… I only knew about this disease before, but I didn’t know exactly what it was…… now I understand it.” (#4).

“…… translates specialized knowledge into something we can understand because we have a limited level of expertise. " (#7).

In addition, participants reported that community-embedded follow-up interventions could help them increase their motivation for health consultation and make health perceptions more concrete. Community-based, focused health education increases the interactivity of the learning process [38].

“…… We can consult directly if we don’t understand something. Because they are professionals, they can tell us exactly what behaviors are good for our health.” (#9).

“This is allowing us to take care of our health. So I will be proactive and ask some questions……” (#3).

Community-embedded follow-up management is considered a behavioral intervention to reinforce health awareness. The participants explained that the benefits they perceived behaviorally would make them more inclined to believe in the cognitive concepts they gained from their learning. This mental reinforcement helped them to be able to make quick and informed decisions and initiatives in their daily lives [39].

### Behavioral outcomes

Participants affirmed that this health service model provided them with a comprehensive care environment that met the service demand.

“…… It’s like a professional senior care service. The difference is that I enjoy comprehensive health services in the comfort of my home. That’s exactly what I need.” (#6).

The participants’ health behavior changes were mainly reflected in the increased initiative of health consultation visits and a healthier daily lifestyle based on the follow-up observations [40]. They believed that this health management intervention helped them to reduce to some extent the pain caused by the disease and to avoid risk factors:

“…… I know how to protect myself from avoiding aggravating my condition by falling.” (#8).

### Psychological care

Community-embedded follow-up management increases the social connection of the elderly population living alone [41]. Participants valued the psychological care provided by this follow-up management model. They indicated that spiritual care reduced the psychological feelings of loneliness and despair:

“…… regularly asked me how I was doing and cared about my life…… they were like a family to me.” (#1).

Participants report that they are more confident and have a sense of dignity when in a good emotional state [42]. They are more willing to proactively engage in self-care behaviors to optimize their quality of life.

“I feel happy under their caring care. I would prefer to become more valuable through my efforts.” (#10).

“…… understands my needs and respects and supports me. With their guidance, I can make my own choices to promote my health.” (#17).

## Discussion

### Summary

This mixed-methods study validated the effectiveness of a community-embedded follow-up management model applied to the health care of older adults with osteoporosis living alone. Participants had positive attitudes towards providing embedded follow-up management services in the community. The quantitative study showed that the scores of physical functioning, ability to perform daily activities, self-efficacy, and psychological status were significantly higher (*P* < 0.05) in elderly osteoporosis patients who received community-embedded follow-up management services. There was a statistically significant difference between the scores of the intervention group and control groups’ scores in each dimension (*P* < 0.05). The qualitative study reported essential components of a community healthcare delivery model for elderly osteoporosis patients living alone, including professional support, personalized services, social support, and empowerment.

### Comparison with existing literature

Consistent with the pre-designed philosophy of this model, professional support is an essential component of the community health services for older osteoporosis patients living alone [43]. Health education and individual coaching by professionals have a significant impact at the level of improving patients’ health awareness and self-care skills [44]. Although most studies on geriatric health care services illustrate this in their reports, the present study identifies the potential influence of professional support in cognition and behavior in a qualitative study. Patients can perceive the favorable effects of achieving self-care with professional guidance. This positive feedback result further reinforces health perceptions and behaviors, implying that lack of professional support may be the root cause of difficulties in achieving effective self-care in older osteoporosis patients living alone [45, 46].

In this intervention management model, community follow-up with interventionists and peer support are critical for older patients living alone to maintain social connections [47, 48]. The research hypothesis that a familiar community environment contributes to the improvement of social behavior was validated in this study [49]. Although Guimaraes et al. reported that prolonged social isolation can lead to a reduction in the response to social behaviors, the present study observed an improvement in socially isolated behaviors and adverse psychological moods such as anxiety in older adults living alone [38]. Consistent with other studies, the effectiveness of peer support applied at the community level to improve psychosocial well-being is generally recognized [38, 50]. The significant value of peer support in facilitating aging in place for community-dwelling older adults was reported in a cohort study by Jacobs et al. The report also encourages the exploration of specific application strategies in community practice applications, which is echoed in the present study [51].

In addition, the issue of empowerment of the elderly has received widespread attention at the primary care level in older communities [52]. As Wong et al. describe in their report, empowering more senior people to function in self-care is essential for promoting a sense of dignity and independence [11, 53]. Most participants wanted to increase their sense of worth through self-care to participate in senior health care services with dignity [54, 55].

### Implications for practice

Future community-wide geriatric health services could adopt an embedded health management model that increases the involvement of community health workers [56]. The elderly living alone suffer from both the frailty associated with osteoporosis and poor psychological outcomes due to lack of social support, and this group should be made a priority focus of community-based geriatric health management services [9]. The professionalism of health education and health care guidance should be strengthened in community-based primary care services to support the realization of an effective self-care process for elderly patients with osteoporosis [57]. A comprehensive health assessment and individualized intervention model is necessary to develop an embedded follow-up management plan [58]. On the one hand, community health workers can assess the elderly population living alone, which is easily neglected in geriatric primary care, and achieve synchronized updating of electronic health records in the community healthcare system [59]. On the other hand, individual differences are fully considered to meet the health needs of elderly patients living alone to a greater extent [60]. For example, the mobilizing factor of peer support is strengthened in health education. This input can be used as an incentive and guidance mechanism to strengthen health cognition and, at the same time, enhance the social connection of elderly osteoporosis patients living alone [38]. The function of elderly patients living alone to perform self-care needs to be preserved in the process of embedded follow-up management interventions in the community, focusing on the issue of elderly empowerment [55]. The degree of intervention can be determined by combining the personal wishes and dignity assessment of elderly patients living alone [61]. In addition, the effective implementation of community-embedded follow-up management requires close collaboration among multidisciplinary professionals. Education on professional training should be provided to the local community health professionals and community workers involved [62].

### Strengths and limitations

The main strength of this mixed study is to assess the effectiveness of a community-embedded follow-up management model applied to the health care of elderly patients with osteoporosis living alone from the perspective of the beneficiaries of health services. The research team rigorously collected quantitative and qualitative data to ensure the comprehensiveness and authenticity of the data. Based on the objective quantitative assessment, the qualitative study explains and adds important components about this community follow-up management model, from which the experience of adequate intervention support for community-embedded follow-up management is summarized to help build a better community health service model.

The self-subjective reports used in this study were not rigorously quantified, so the results may be biased, which will be further improved in future studies. In addition, the diversity of geriatric comorbidities may limit the effectiveness of disease-specific interventions. It should also be noted that the population interviewed in this study was mainly in the community of Nantong, Jiangsu Province. At the same time, other issues may be discussed in different regions. Our data need to be investigated and confirmed in future studies with broader populations, and we look forward to related studies by other research groups.

## Conclusions

Overall, community-embedded follow-up management meets the need for health services for elderly patients with osteoporosis living alone. It helps improve health awareness, promote physical and mental health, and optimize the quality of life in this population. Personalized services and professional support are the two main contributing factors to effective embedded follow-up management in the community.

### Electronic supplementary material

Below is the link to the electronic supplementary material.

Supplementary Material 1

## Data Availability

The datasets used and analyzed during the current study are available from the corresponding author on reasonable request.

## Abbreviations

HPA: Hypothalamic-pituitary-adrenocortical
FLS: Fracture Liaison Service
CHWs: Community Health Workers
CGA: Comprehensive Geriatric Assessment
WHO: World Health Organization
DAX: Dual-energy X-ray absorptiometry
IOF: International Osteoporosis Foundation
VAS: Visual analogue scale
