# Supplementary material for: Community-embedded follow-up management intervention for geriatric primary care: a mixed-methods study of an integrated health services model
Source: BMC Health Serv Res. 2024 Mar 6;24:298. doi: 10.1186/s12913-024-10804-8 (PMC10918903; doi:10.1186/s12913-024-10804-8)
Supplement: Supplementary file 1 — Supplementary Material 1 [file 12913_2024_10804_MOESM1_ESM.docx]

**Appendice**

**Interview guide (English translation)**

**Presentation**

Dear Sir/Madam:

First, thank you very much for participating in this 6-month Community Embedded Follow-up Health Management Service project and for allowing me to interview you. My name is XXX, and I am the person responsible for interfacing with you to interview for this project. I would like to know the impact of this community-embedded follow-up health management service model on the daily lives of elderly osteoporosis patients who live alone. By talking to you, I can gain a better understanding of this issue. Therefore, thank you very much for your willingness to spend time with me.

I want to gain a more in-depth understanding of your views and experiences of the community-embedded follow-up management service you receive in your daily life, so there are no right or wrong answers, which means you are welcome to tell us what you are sure of about the model and if there is anything that needs to be improved.

Before the formal interview, I would like you to explain a few things about this interview. The interview will take no more than 40 minutes, depending on how long we talk. This interview is voluntary, and you may withdraw anytime for any reason. In addition, the content of the interview has been anonymized(1). Therefore, if I share something from the interview with my colleagues, they will not know who said it.

Finally, to better focus on our conversation, I would like to ask if I can record our interview. I will not share the recording with anyone, and I will delete the recording after taking notes.

The interviews were structured as follows(2):

- General characteristics
- Your experience of receiving community-embedded follow-up management services
- Your intended goals, perceptions, and recommendations
- Closing with a thank you and data use statement

**General Characteristics(2)**

1. How old are you?
2. How do you feel about your health? Are there any other underlying diseases? Does it affect your daily activities?
3. Can you describe your daily life to me?

**Experiences**

1. Can you describe how the Community Embedded Follow-up Management Health Service has been used in your daily life over the past six months? To what extent has it been used?(3, 4)
2. Has your life changed due to receiving the Community Embedded Follow-Up Managed Health Service? How has it changed? Do you think the changes have been beneficial? Why? (Try to ask about different aspects, for example, diet, exercise, health perceptions, health behaviors, psychological status, self-perceived health status, etc.)(3, 5)

**Goals, Perceptions, and Suggestions**

1. Do you expect health support through community health services? In what ways do you wish to be helped? Are there any specific examples(6, 7)?
2. Are you satisfied with the community-embedded follow-up management health services you have received in the past six months? Can you be specific about what made you feel satisfied? Why?(8-10)
3. What are the strengths of the community-embedded follow-up management health service? What are the disadvantages?(10, 11)
4. Are there any areas for improvement in this community-embedded follow-up management health service?(7, 12)

**End**

This is the interview's end. Do you have anything to add before we finish? If not, thank you very much for accepting my interview. If you have any other thoughts or questions, please get in touch with me.

Here is what I will do to process the data: I will process and summarise the data. If you wish, I can send it to you for review. I can get their contact information now if you would like to recommend someone else.

Have a nice day!

XXX

1. Husband G. Ethical Data Collection and Recognizing the Impact of Semi-Structured Interviews on Research Respondents. Education Sciences. 2020;10(8).

2. Kallio H, Pietilä AM, Johnson M, Kangasniemi M. Systematic methodological review: developing a framework for a qualitative semi-structured interview guide. Journal of Advanced Nursing. 2016;72(12):2954-65.

3. Roberts RE. Qualitative Interview Questions: Guidance for Novice Researchers. Qualitative Report. 2020;25(9):3185-203.

4. Beechem MH, Anthony C, Kurtz J. A life review interview guide: A structured systems approach to information gathering. International Journal of Aging & Human Development. 1998;46(1):25-44.

5. Zoffmann V, Hörnsten Å, Storbækken S, Graue M, Rasmussen B, Wahl A, et al. Translating person-centered care into practice: A comparative analysis of motivational interviewing, illness-integration support, and guided self-determination. Patient Education and Counseling. 2016;99(3):400-7.

6. Langlois S, Goudreau J. "From Health Experts to Health Guides": Motivational Interviewing Learning Processes and Influencing Factors. Health Education & Behavior. 2022.

7. Kraun L, van Achterberg T, Vlaeyen E, Fret B, Briké SM, Ellen M, et al. Transitional care decision-making through the eyes of older people and informal caregivers: An in-depth interview-based study. Health Expectations. 2023;26(3):1266-75.

8. Wright J, Moghaddam N, Dawson DL. Cognitive Interviewing in Patient-Reported Outcome Measures: A Systematic Review of Methodological Processes. Qualitative Psychology. 2021;8(1):2-29.

9. Kumar P, Follen M, Huang CC, Cathey A. Using Laddering Interviews and Hierarchical Value Mapping to Gain Insights Into Improving Patient Experience in the Hospital: A Systematic Literature Review. Journal of Patient Experience. 2020;7(6):1740-7.

10. Olsen M, Udo C, Dahlberg L, Boström AM. Older Persons' Views on Important Values in Swedish Home Care Service: A Semi-Structured Interview Study. Journal of Multidisciplinary Healthcare. 2022;15:967-77.

11. Fridberg H, Wallin L, Tistad M. The innovation characteristics of person-centred care as perceived by healthcare professionals: an interview study employing a deductive-inductive content analysis guided by the consolidated framework for implementation research. Bmc Health Services Research. 2021;21(1).

12. Kelly A, Tymms K, Fallon K, Sumpton D, Tugwell P, Tunnicliffe D, et al. Qualitative Research in Rheumatology: An Overview of Methods and Contributions to Practice and Policy. Journal of Rheumatology. 2021;48(1):6-15.
